# Traumatic Events and Cognitive Function: Does Time When Traumatic Events Happen Matter?

**DOI:** 10.1093/geroni/igab046.1653

**Published:** 2021-12-17

**Authors:** Gabriella Dong

**Affiliations:** Rutgers, The State University of New Jersey, New Brunswick, New Jersey, United States

## Abstract

People at different life stage may respond differently to traumatic events and result in different cognitive health. This study aims to examine the relationship between life stage at which one experiences traumatic events and cognitive function. The data were drawn from the 2017-2019 PINE study (N = 3,125). The time of life events happened was evaluated by childhood (<20), adulthood (20-59), and old age (60 and above). Cognition was measured through global cognition, episodic memory, working memory, processing speed, and MMSE. Linear regression was used. Individuals with the latest exposure to traumatic events at adulthood or old age have higher cognitive function than those without traumatic events over the life course. Exposure to traumatic events in middle or later life stimulates cognition, while trauma exposure in earlier life stage does not. Future research to understand the impact of traumatic events on health could consider the time when traumatic events happen

